# Household

**Published:** 1889-10

**Authors:** 


### HOUSEHOLD.

MAGIC HAIR DRESSING.—To ½ gallon of soft water, add ¾ lb. of glycerine, ½ lb. tincture of cantharides, ¼ oz. oil of jasmine, ⅛ oz. oil of verbenia, ½ oz. essence of emon, and 1 oz. essence of bergamot. Let it stand twenty-four hours and it is fit for use. This dressing keeps the hair soft, moist and glossy, but it is not oily or greasy, nor will the hair dressed with it soil the finest linen. This is regarded as the most valuable receipt now known for a tonic hair dressing. It is confidently recommended to keep the hair from falling in nine cases out of en. At first it should be used daily, say for a week or ten days, then every alternate day. The head should be gently washed in warm soap-suds—castile soap is preferable—once in two weeks, to keep the scalp in a clean and healthy condition.

VELVET CAKE.—Beat the yolks of six eggs until frothy. Add two cups of granulated sugar, and beat for fifteen minutes. Add the well-beaten whites of three eggs, leaving the other three for the frosting. Stir into one cup of boiling water 2½ cup of flour, with one tablespoonful of baking powder mixed dry. Flavor with one tablespoonful of lemon extract. Bake in three layers, and put this icing between them. The whites of three eggs, well beaten, six dessertspoonfuls of pulveried sugar to each egg. Beat stiffi, and flavor with lemon.

PANSIES ALL THE YERR ROUND.—Among flowers which we cannot bear to give up when their season's cup of beauty is drained, but which we long to have abide with us, to admire their beauty and enjoy their companionship all the year round, the pansy is peerless, and very kindly it lends itself to ways and means for perpetuating its beauty.

Large, well formed, well colored blooms are, perhaps, more difficult to obtain during the heat of summer than at any other time ; but the pansy fancier who will plant summer bloomers—from seed sown the autumn beforehand—in a bed cut in the lawn on its northern exposure, where there is partial shade, and who is willing to water copiously every evening during dry weather, giving stimulants once a week, will scarcely fail to secure the coveted two-inch blooms during even the hottest weather.—*Vick's Magazine* for July.

Remove flower-pot stains from window sills by rubbing with fine wood ashet and rinse with clear water.

Washing pine floor in a solution of one pound of copperas dissolved in one gallon of strong lye gives oak color.

Stains on ivory may be taken out by washing with soap and wa ter and placing it while wet, in the air to bleach.

If matting, counterpanes, or bed-spreads have oil spilt on them, wet with alcohol, rub with hard soap, and then rise with clear cold water.

To take ink stains out of table-cloths, napkins, etc., put the article to soak imme diately in thick sour milk, changing the milk as often as necessary.

Wash hair brushes and combs in soft water and liquid ammonia in the proportion of four teaspoonfuls of liquid ammonia to one quart of water.

